# Single left superior vena cava: antenatal diagnosis, associated anomalies and outcomes

**DOI:** 10.1002/uog.24966

**Published:** 2022-11-01

**Authors:** K. R. M. Lopes, M. Bartsota, V. Doughty, J. S. Carvalho

**Affiliations:** ^1^ Brompton Centre for Fetal Cardiology Royal Brompton and Harefield Hospitals London UK; ^2^ Fetal Medicine Unit St George's University Hospitals NHS Foundation Trust London UK; ^3^ Cardiovascular Clinical Academic Group St George's University of London Molecular and Clinical Sciences Research Institute London UK

**Keywords:** congenital heart defect, echocardiography, fetal heart, genetic disease, persistent left superior vena cava, prenatal diagnosis, ultrasonography

## Abstract

**Objectives:**

To describe the associated cardiac and extracardiac findings and estimate the prevalence of single left superior vena cava (LSVC) among fetuses referred for fetal echocardiography.

**Methods:**

This was a retrospective case series of fetuses diagnosed with situs solitus and single LSVC at the Brompton Centre for Fetal Cardiology, London, UK, from October 2006 to December 2020. Prenatal and postnatal outcome data were collected. Prenatal diagnosis was based on abnormal vessel alignment at the three‐vessel view and/or three‐vessel‐and‐trachea view, showing a vessel to the left of the pulmonary artery (i.e. the LSVC) and absence of the usual vessel to the right of the ascending aorta (i.e. the right superior vena cava), and further visualization of the LSVC draining into the coronary sinus.

**Results:**

Of 19 968 fetal echocardiograms performed during the study period, 34 cases of single LSVC were identified (a prevalence of 0.17%). Of these, 32 pregnancies had a live birth, one was lost to follow‐up and one resulted in intrauterine demise. Single LSVC was isolated in 79.4% of cases. No major congenital heart disease was identified. One fetus showed mild isthmus hypoplasia, with no aortic coarctation postnatally. Two fetuses had umbilical vessel abnormalities. A genetic abnormality was found in one case (15q24.1‐q24.2 deletion).

**Conclusions:**

Antenatal diagnosis of single LSVC in the setting of situs solitus is usually a benign isolated finding. Nevertheless, investigation of other cardiac, extracardiac and genetic disorders should be considered. © 2022 The Authors. Ultrasound in Obstetrics & Gynecology published by John Wiley & Sons Ltd on behalf of International Society of Ultrasound in Obstetrics and Gynecology.

**CONTRIBUTION**

**What are the novel findings of this work?**

This is the largest series of single left superior vena cava (LSVC) diagnosed antenatally. In the majority of cases, this was an isolated finding. No major cardiac abnormalities were seen, although significant extracardiac or genetic findings were seen in ∼6% of cases. Most fetuses with a single LSVC were identified after introduction of the three‐vessel and three‐vessel‐and‐trachea views into routine screening despite the four‐chamber view also being abnormal.

**What are the clinical implications of this work?**

More fetuses with single LSVC are likely to be detected as antenatal screening improves. In contrast to fetuses with bilateral superior vena cava, significant associated abnormalities are uncommon in fetuses with single LSVC. However, it is still important to perform a thorough investigation for possible associated abnormalities and consider genetic disorders, despite an expected low yield.

## INTRODUCTION

Anomalies of the venous system are common. With the increasing implementation of prenatal screening for malformations, more cases of venous anomalies are diagnosed antenatally. During embryogenesis, the systemic venous circulation includes the right and left anterior cardinal veins; the right anterior cardinal vein evolves to form the right superior vena cava (RSVC) and the bridging innominate vein, while the left anterior cardinal vein regresses. Sporadically, the left anterior cardinal vein fails to regress, resulting in the formation of a persistent left superior vena cava (LSVC), which usually drains into an enlarged coronary sinus. The majority of individuals with LSVC have an intact RSVC, namely, bilateral superior vena cava (BSVC). This is the most common systemic venous anomaly, with a reported incidence of 0.2–0.5% in low‐risk fetal populations^1^, ^2^, ^3^, reaching 9% in fetuses with congenital heart disease (CHD)^2^.

In rare circumstances, the right anterior cardinal vein regresses, resulting in absence of the RSVC and a persistent LSVC, namely, single LSVC. In such cases, the entire upper body venous drainage is through the single LSVC, which usually drains into the enlarged coronary sinus. This diagnosis is usually reported as an incidental finding during invasive procedures or autopsy series, with a reported incidence of 0.05%^4^, ^5^, ^6^. Only a few, isolated cases of antenatal diagnosis of single LSVC have been reported to date^7^, ^8^, ^9^, ^10^, ^11^, ^12^.

Herein, we present a series of 34 consecutive cases of single LSVC diagnosed prenatally and describe the associated cardiac and extracardiac findings and estimated prevalence in a selected population of fetuses referred for fetal echocardiography.

## METHODS

This was a retrospective study of a cohort of fetuses diagnosed with single LSVC at a tertiary center. The aim was to describe the features and frequency of antenatal diagnosis, associated anomalies and outcomes of single LSVC to help with prenatal counseling. Cases were identified from our fetal echocardiography database at the Brompton Centre for Fetal Cardiology, London, UK, from our first diagnosis in October 2006 until December 2020. The study was registered as a clinical audit (004398) and no ethical approval was required, in accordance with local governance.

The inclusion criterion was the presence of a single LSVC in the setting of situs solitus. Cases associated with isomerism were excluded, since absence of the RSVC is a common feature of heterotaxy syndrome. In this paper the term ‘single LSVC’ implies the presence of situs solitus. Pre‐ and postnatal data were retrieved from medical charts and hospital clinical databases. Antenatal data included maternal age, reason for referral, gestational age at diagnosis, cardiac and extracardiac findings and karyotyping or other genetic studies. Postnatal cardiac, pediatric and genetic follow‐up data were systematically collected, including data from outreach clinics if the child was followed up elsewhere.

Fetal echocardiography comprised a comprehensive cardiovascular examination by experienced fetal cardiologists using various ultrasound systems (Aloka Alpha 10 (Aloka Medical, Ltd, Tokyo, Japan); Aplio i800 (Canon Medical Systems Inc., Tokyo, Japan); Voluson E10 (GE Healthcare, Zipf, Austria)) with a curvilinear transducer of appropriate frequency for the patient and gestational age. The cardiac scans were carried out in a segmental approach, combining two‐dimensional imaging, M‐mode and color/pulsed‐wave Doppler imaging. Systemic, pulmonary and umbilical venous return and the great arteries are systematically evaluated in all fetal echocardiograms in our unit, and information is entered routinely in the appropriate computer database at the time of scanning. When required, additional information was retrieved from recorded videoclips and stored images.

**Figure 1:**
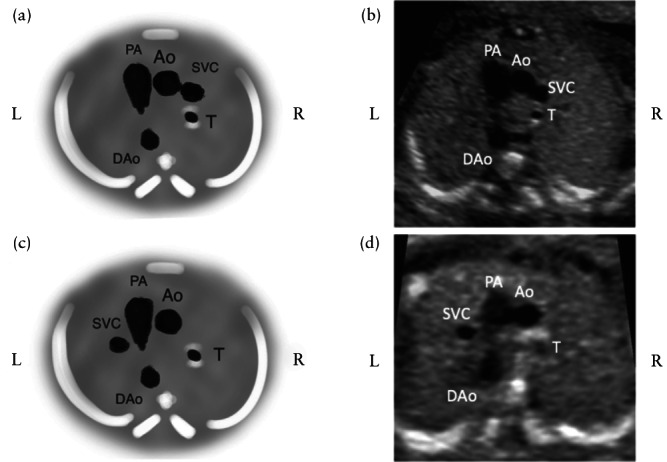
Schematic diagrams (a,c) and ultrasound cross‐sectional images (b,d) of the three‐vessel view showing: (a,b) normal arrangement of the vessels, with the pulmonary artery (PA), aorta (Ao) and right superior vena cava (SVC) seen from left to right, in a fetus with normal cardiac anatomy; (c,d) abnormal arrangement of the vessels in the setting of single left SVC, with the left SVC, PA and Ao seen from left to right. DAo, descending aorta; L, left; R, right; T, trachea.

**Figure 2:**
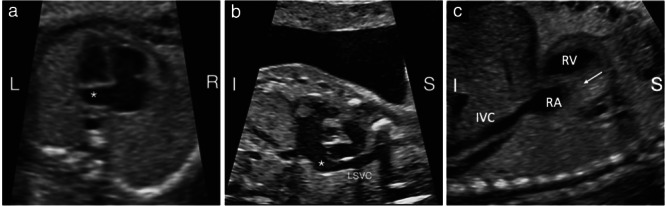
Ultrasound images from different fetuses in midgestation with single left superior vena cava (LSVC). (a) Apical cross‐section of the four‐chamber view in a slightly caudal plane demonstrating the dilated coronary sinus (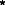). (b) Sagittal‐oblique view of the LSVC showing its drainage into the coronary sinus (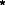). (c) Bicaval view demonstrating the inferior vena cava (IVC) draining normally into the right atrium (RA) and absence of the right superior vena cava (arrow). I, inferior; L, left; R, right; RV, right ventricle; S, superior.

A prenatal diagnosis of single LSVC was made on the basis of an abnormal three‐vessel view (3VV) and/or three‐vessel‐and‐trachea view (3VTV) at the upper mediastinum, showing the presence of a vessel to the left of the pulmonary artery, i.e. the LSVC, together with the absence of the usual vessel to the right of the ascending aorta, i.e. the RSVC (Figure 1). In addition, on color Doppler, the presence of reversed flow direction at the innominate vein, from right to left, should raise the suspicion of single LSVC. The diagnosis was then confirmed in the long‐axis view, demonstrating the site of drainage of the LSVC into the dilated coronary sinus and the absence of the RSVC in the bicaval view (Figure 2). In the four‐chamber view, the coronary sinus was dilated.

All parents received detailed counseling after the fetal cardiac scan. Extracardiac structures were evaluated by experienced practitioners or fetal medicine specialists. Invasive genetic prenatal diagnosis, i.e. karyotype/microarray‐based comparative genomic hybridization (array‐CGH), or non‐invasive prenatal testing (NIPT) were not offered systematically. Follow‐up scans were arranged, usually one further scan during the third trimester to document normal growth of cardiac structures and review the aortic arch, and a perinatal management plan was established accordingly. It is our policy to organize postnatal follow‐up for any cardiac abnormality detected antenatally. This is to confirm normal transition from fetal to postnatal circulation (closure of the foramen ovale and arterial duct), verify coronary artery anatomy and to reassess for minor defects that can potentially be overlooked prenatally. Additionally, in the absence of formal genetic investigations, postnatal follow‐up allows confirmation of phenotype.

Descriptive statistics are reported as median (range) or median (interquartile range (IQR)) for continuous variables and as *n* (%) for categorical variables. Statistical analysis was performed using Microsoft Excel, version 16.42 (Microsoft, Redmond, WA, USA).

## RESULTS

Of 19 968 fetuses examined by echocardiography between October 2006 and December 2020, a total of 34 (0.17%) were diagnosed with single LSVC. The median gestational age at diagnosis was 23 + 4 weeks (range, 17 + 6 to 33 + 5 weeks). Maternal age ranged from 17 to 46 years (median, 32 (IQR, 29.5–36.5)).

**Figure 3:**
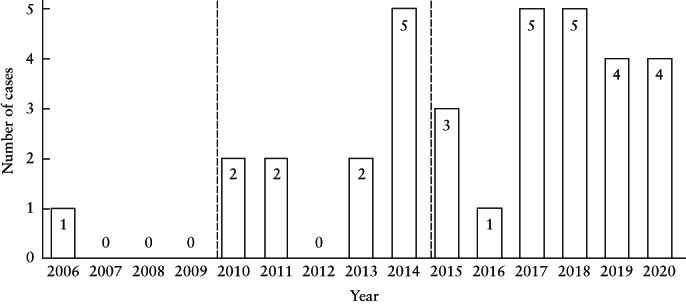
Frequency distribution of cases with single left superior vena cava diagnosed over the study period. The two dotted lines indicate the year of introduction of the three‐vessel view (2010) and three‐vessel‐and‐trachea view (2015) into the cardiac screening protocol in England.

The main reason for referral was suspected cardiac abnormality at the routine anomaly scan, accounting for approximately 85% of cases, followed by increased nuchal translucency thickness in the remaining cases (Table 1). The frequency distribution of these referrals over the study period is shown in Figure 3.

**Table 1:** Antenatal and postnatal findings and outcome of 34 fetuses diagnosed prenatally with single left superior vena cava

Parameter	Value
Reason for referral	
Abnormal 3VV/3VTV	24 (70.6)
Abnormal four‐chamber view	5 (14.7)
Increased nuchal translucency	5 (14.7)
Other fetal cardiac findings	
Small muscular VSD	2 (5.9)
Normal aorta	33 (97.1)
Isthmal hypoplasia	1 (2.9)
Other postnatal cardiac findings*	
Small coronary fistula and bronchial collaterals	1/30 (3.3)
Isolated bronchial collaterals	1/30 (3.3)
Extracardiac findings	
Single umbilical artery	1 (2.9)
Umbilical vein varix, FGR, ventriculomegaly, cardiomegaly	1 (2.9)
Genetic findings†	
15q24 deletion syndrome	1/32 (3.1)
Outcome	
Intrauterine death	1 (2.9)
Lost to follow‐up	1 (2.9)
Pending postnatal review	2 (5.9)
Live birth with postnatal follow‐up	30 (88.2)

Data are presented as *n* (%) or *n*/*N* (%).

* Data reported only for 30 cases with known outcome data.

† Data reported only for 32 cases that had genetic testing and/or with known phenotype. 3VV, three‐vessel view; 3VTV, three‐vessel‐and‐trachea view; FGR, fetal growth restriction; VSD, ventricular septal defect.

In four cases (11.8%), the absence of the RSVC was overlooked on the first fetal echocardiogram, but was appreciated on subsequent scans. Of the 34 cases, one was lost to follow‐up, one resulted in spontaneous intrauterine death and two liveborn infants were awaiting postnatal review at the time of writing. Therefore, postnatal follow‐up and confirmation of the diagnosis were available for 30/34 cases. There was no case of termination of pregnancy. The survival rate was 97.1% (33/34). Single LSVC was isolated in the majority (79.4%) of fetuses.

### Cardiac findings

No major structural CHD was identified prenatally, but two fetuses showed a small muscular ventricular septal defect. Postnatally, there were additional minor echocardiographic findings in two children. Each had small bronchial collaterals, one of whom also had a small coronary fistula to the pulmonary artery (Table 1). These were of no hemodynamic significance and required no treatment. The aortic arch was normal in all cases, with no evidence of discrete coarctation. One fetus showed mild isthmal hypoplasia but a normal aorta postnatally.

### Extracardiac and genetic findings

Extracardiac findings were present in two (5.9%) fetuses. One had a single umbilical artery, normal NIPT and normal phenotype. One growth‐restricted fetus, which presented with cardiomegaly, had a large umbilical vein varix, mild cerebral ventriculomegaly and normal array‐CGH. This fetus died *in utero* at 32 weeks' gestation, probably owing to heart failure secondary to large umbilical vein varix.

Genetic investigation was carried out in approximately one‐quarter (26.5%) of the cases. Specifically, polymerase chain reaction and array‐CGH were performed in five fetuses and one child (17.6%) and cell‐free DNA testing in three (8.8%) cases. Results were normal in all but one case, in which a 15q24.1‐q24.2 deletion, which overlaps 15q24 deletion syndrome, was identified postnatally as part of investigations for mild neurodevelopmental delay.

## DISCUSSION

We describe a cohort of 34 fetuses with single LSVC – the largest reported cohort diagnosed antenatally – and show this to be an isolated condition in the majority (79.4%) of cases. Only two (5.8%) fetuses had abnormalities with clinical impact, one extracardiac and one genetic. Major CHD was not present in any case. Minor cardiac findings were identified in four (11.8%) cases. The aortic arch was normal in all fetuses.

Only 13 cases of single LSVC have previously been reported prenatally, which were isolated^7^, ^8^, ^9^, ^10^, ^12^, with the exception of one case that was associated with VACTERL^11^. However, in postnatal series of single LSVC, there is a stronger association with CHD (46–100%)^4^, ^5^, ^6^, and some cases also had rhythm (25%) or chromosomal abnormalities, including trisomy 21 and trisomy 18 (7%)^5^. The postnatal diagnosis of isolated single LSVC is usually an incidental finding at autopsy or on invasive procedures^5^, ^6^.

The rate of single LSVC in fetuses referred for fetal echocardiography in this study (0.17%) is about three times that reported in general postmortem series (0.05%)^4^ and twice that seen in series of CHD^5^. Our estimated prevalence is presumably closer to that of the general population, since single LSVC can be suspected during routine screening that incorporates the 3VV and 3VTV. Although all cases described in this study underwent fetal echocardiography, ∼85% were from low‐risk families. However, the true prevalence may be higher, as it is still possible that not all cases will be picked up by screening.

In the UK, The Fetal Anomaly Screening Programme^13^ is part of the wider screening program of the National Health Service. It includes a detailed fetal structural investigation, usually performed by trained sonographers. A national cardiac protocol was introduced in 2010 that included the 3VV, and in 2015 the 3VTV was added^14^. These screening cardiac views are in agreement with international guidelines^15^. It is of interest to note that, in our study, only one case was diagnosed prior to 2010 and the majority (64.7%) were detected since 2015 (Figure 3). Our data point to a clear increase in the detection of single LSVC with the incorporation of the 3VV and 3VTV in routine screening protocols. In these views, three vessels will be present but ‘abnormally’ arranged, as shown in Figures 1 and 2.

An abnormal 3VV and/or 3VTV on routine screening was the main reason for referral in cases in which an abnormality was suspected (24 of 29 cases), followed by an abnormal four‐chamber view (five of 29 cases). It is interesting that, although, in the 29 cases, the four‐chamber view was abnormal owing to a dilated coronary sinus, which receives the entire upper body blood return, this was not the main reason for referral.

It has been postulated that dilatation of the coronary sinus could lead to restriction of left ventricular inflow, contributing to the development of left heart obstructive disease^16^. However, a recent meta‐analysis concluded that BSVC, a condition associated with dilated coronary sinus, did not carry an increased risk for coarctation^17^. In this series, no coarctation or left heart obstruction was present.

**Figure 4:**
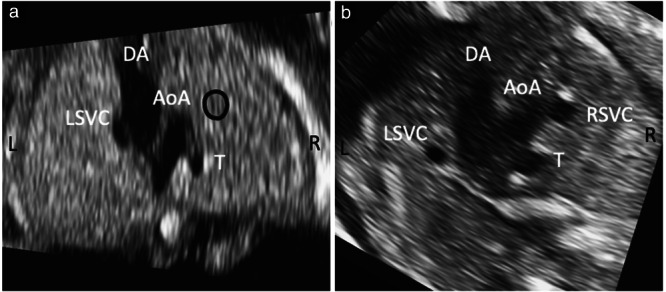
Ultrasound cross‐sectional images at the level of the three‐vessel‐and‐trachea view in a case with single left superior vena cava (LSVC) (a) and a case with bilateral superior vena cava (b). Note that in (a), the fluid‐filled space to the right of the aorta corresponds to the trachea (T), which should not be mistaken for the right superior vena cava (RSVC). The trachea shows an echogenic ring and occupies a more posterior position than that expected for the RSVC, the absence of which is indicated by the black circle. AoA, aortic arch; DA, ductus arteriosus; L, left; R, right.

BSVC is a relatively frequent antenatal finding, therefore when persistent LSVC is identified during a fetal heart scan, the RSVC will be present in the vast majority of cases. However, one should not assume this is the case, as it is necessary to visualize both superior vena cavas. In four of our cases, absence of the RSVC was not appreciated on the first scan. Care should be taken to identify correctly the upper mediastinal structures and not to misinterpret the trachea as being the RSVC (Figure 4). The trachea is more posterior to the normal position of the RSVC and has a hyperechogenic ring. In addition, in BSVC, the bridging innominate vein is usually absent, whereas in single LSVC it is present, but flow direction is right to left, i.e. the opposite to normal.

LSVC draining into the coronary sinus, whether single or bilateral, is a venous abnormality with no hemodynamic significance but with different clinical implications. BSVC is frequently associated with cardiac or extracardiac findings^3^, ^18^, ^19^, ^20^. When both are present, genetic abnormalities are reported in up to 45% of cases^19^. When BSVC is strictly isolated, a genetic anomaly is usually absent. Conversely, single LSVC is usually an isolated antenatal finding^7^, ^8^, ^9^, ^10^, ^12^, as seen in this series. Genetic abnormalities are rarely reported in single LSVC^11^, and such an abnormality was seen in only one case in our series. The association between single LSVC and cardiac, extracardiac and genetic disorders seems to be low, but not absent (Table 2).

**Table 2:** Prevalence of single left superior vena cava (SVC) and bilateral SVC and presence of associated anomalies, based on the largest published antenatal series of persistent left SVC

	Single left SVC: this series	Bilateral SVC	
		Minsart (2020)^19^, *	Du (2014)^3^, *
*n*	34	229	164
Prevalence	0.17	1.0	0.7
Isolated	79.4	17.0	26.8
Associated abnormality			
Cardiovascular only	11.8	31.4	19.5
Extracardiac only	5.8	12.7	17.7
Cardiovascular and extracardiac	0	38.9	27.4
Genetic	2.9	28.4	15.4†

Data are given as %, unless stated otherwise. Only first author of each study is given.

* Includes cases of abnormal situs/heterotaxy.

† Confirmed by karyotype.

It is now commonly accepted that, in fetuses with an increased nuchal translucency thickness, irrespective of the karyotype, the prevalence of major CHD is higher than in the general population, although the mechanism underlying this relationship is still poorly understood^21^. Increased nuchal translucency has been reported in 29% of fetuses with BSVC^2^, whereas in this series it was increased in about 15% of cases.

This study, despite being retrospective, reports the largest series of single LSVC diagnosed antenatally. The use of a standardized protocol for fetal heart screening in our network, with systematic referral for fetal echocardiography in the presence of abnormal cardiac views, is one of the strengths of the study. Similarly, our unit's strict protocol for postnatal follow‐up of any cardiac abnormality detected antenatally allowed us to have good postnatal outcome data. Although the number of genetic studies performed was relatively low, we were able to confirm normal phenotype in the vast majority of cases.

In conclusion, when single LSVC is diagnosed antenatally it is usually a benign isolated finding. Nevertheless, it is important that these cases be thoroughly investigated to exclude associated cardiac, extracardiac and genetic disorders, even if the risk appears to be small. With the increasing implementation of prenatal screening for malformations and improvement in screening standards, more cases of single LSVC are likely to be detected prenatally.

## Data Availability

The data that support the findings of this study are available from the corresponding author upon reasonable request.
